# HIV prevention: What have we learned from community experiences in concentrated epidemics?

**DOI:** 10.1186/1758-2652-11-5

**Published:** 2008-10-01

**Authors:** Bruno Spire, Isabelle de Zoysa, Hakima Himmich

**Affiliations:** 1 INSERM, U912 (SE4S), Marseille, France; 2 Université d'Aix Marseille, Marseille, France; 3 AIDES association, Pantin, France; 4 World Health Organisation, Geneva, Switzerland; 5 Association de lutte contre le sida, (ALCS), Casablanca, Morocco

## Abstract

Drawing on lessons learned from community experiences in concentrated epidemics, this paper explores three imperatives in the effort to reduce the sexual transmission of HIV: combat prevention fatigue, diversify HIV testing and combat stigma and discrimination. The paper argues for a non-judgmental harm reduction approach to the prevention of sexual transmission of HIV that takes into account the interpretation of risk by diverse individuals and communities in the era of antiretroviral therapy. This approach requires greater attention to increasing access to opportunities to know one's serostatus, especially among key populations at greater risk. Novel approaches to diversifying HIV testing approaches at community level are needed. Finally, the paper makes a plea for bold measures to combat stigma and discrimination, which continues to represent a formidable barrier for access to services for affected populations and may contribute to HIV-related risk behaviours. A "triple therapy" approach to address stigma and discrimination is discussed, which includes greater acceptance of people living with HIV and AIDS (PLWHA), improving relevant laws and policies, and involving prevention users- *working with people rather than for people-*.

Note: this paper corresponds to the plenary talk of Bruno Spire at the XVIIth World AIDS Conference, August 8th, Mexico city: http://www.kaisernetwork.org/health_cast/player.cfm?id=4383

## 

Although about 20 antiretroviral medications have now been approved for treating people living with HIV and AIDS (PLWHA), only a limited number of proven HIV prevention tools and interventions are available, while others are still at the research stage [1]. An example of a newly proven intervention to reduce HIV risk is male circumcision [2]. Male circumcision, however, will not fulfil all prevention needs [3]. The challenge is to scale-up comprehensive HIV prevention programmes based on all approaches that are known to work. For instance, there is abundant evidence that harm reduction, which includes needle syringe programming and access to opioid substitution therapy, reduces HIV transmission among injecting drug users [4,5]. It is also known that interventions promoting safer sex, in particular through condom use, can reduce sexual transmission of HIV [6].

However, a narrowly conceived "ABC" strategy has limitations. Abstinence-only programs do not work [7], and a more comprehensive and balanced approach is required that addresses different life stages and situations. There is evidence that comprehensive behavioural interventions can result in delayed entry into sexual life and reduced HIV incidence among young people [8]. Interventions promoting partner reduction, or discouraging concurrency among those who are sexually active, are crucial although they have had limited success among key populations at highest risk in most settings [9]. Finally, consistent and correct use of condoms reduces HIV transmission, but is not sufficient to meet everyone's needs [10]. The reality is that lifelong consistent condom use is not acceptable or feasible for most people.

This paper considers lessons learned from community experiences in concentrated epidemics that should serve to improve HIV prevention programming, using tools and interventions that are currently available. It also explores three imperatives in the effort to reduce the sexual transmission of HIV: combat prevention fatigue, diversify HIV testing and combat stigma and discrimination.

## Combating prevention fatigue

### The need for a non-judgmental harm reduction approach to the prevention of sexual transmission of HIV

"Prevention fatigue" is said to pose a threat to the acceleration and sustainability of HIV prevention efforts. However, the prevention discourse is often pitched in "all or nothing" terms, while the concept of progressive risk reduction has not been sufficiently applied. Renewing the discourse on safer sex and adopting approaches that are tailored to the needs of individuals and communities might help to boost flagging programmes.

"Prevention fatigue" has been raised as an issue for the gay community in most industrialised countries [11]. In France, repeated cross-sectional studies carried out among readers of the gay press have documented increases in the rate of unprotected anal intercourse, from 20% in the nineties to 33% in 2007 [12]. Similar relapses in risk behaviour have been observed in other countries [13,14]. The gay community has been widely criticized for insufficient action and continued high levels of unsafe sex. However, a national survey of sexual behaviour carried out in France between October 2005 and March 2006 among more than 12,000 individuals, shows that men who have sex with men (MSM) have more condom use experience than heterosexual populations [15]. In this study, the proportion of people who reported condom use in the last year is higher among MSM than among men who have sex with women (MSW), especially in stable relationships (93% of MSM vs. 81% of MSW with three partners or more in the previous year used condoms, and 53% of MSM vs. 17% of MSW with one partner only in the last year used condoms). This suggests that MSM may achieve equal or higher comfort levels with condoms than heterosexual populations.

Instead of dwelling on the times when so many members of the gay community died from AIDS related illnesses, despite widespread community action and considerable changes in sexual behaviour, it would be more constructive to work at reaching out to, and developing pragmatic solutions for those who, for one reason or another, do not consistently practice safe sex.

Understanding the conditions of risk and how people interpret risk is of key importance when designing appropriate HIV prevention strategies [16,17]. For many people, HIV risk forms part of the fabric of their life. Take the woman who is afraid to ask her husband to use a condom because he might beat her, the young gay man who is learning to enjoy his sexuality and struggling to manage new sexual relationships, or the sex worker who has to deal with clients who refuse to use condoms. These are real life situations in which systematic condom use is just not happening. The issue is how to manage risk in ways that minimize the impact of risky exposures. This is challenging in a society that refuses to accept that people take risks and blames those who do.

A way forward is to adopt a non-judgmental harm reduction approach to the prevention of sexual transmission of HIV. HIV prevention programmes should be designed under the assumption that, with few exceptions, HIV-negative people do not want to get the virus; and that PLWHA do not want to transmit the virus. Our work on the ground shows that one of the greatest concerns of PLWHA is ongoing HIV transmission [18]. *People do care*. Those who take risks also care. Despite the lack of evidence on the effectiveness of this approach, some women use other barrier methods, such as diaphragms, when they cannot use male or female condoms, in the hope of reducing their risk of HIV infection, because these methods are under their control and undetectable by their sexual partner [19].

Among HIV-positive MSM, serosorting is frequently observed [14]. Although it is not demonstrated to be effective for protecting HIV-negative individuals, some individuals already living with HIV are choosing this approach with the goal of unprotected intercourse. MSM have also been observed to adapt their sexual practices through strategic positioning [20]. Among networks of gay men who otherwise seek unprotected intercourse, condoms may be used in the event of sexually transmitted infections [21]. Of course, the effectiveness of these strategies in reducing HIV risk is uncertain. The point is that people who have difficulties with condom use do care at some level about HIV risk, or else they would not bother to use alternative strategies to reduce this risk.

There is some urgency to move beyond the "all or nothing" approach to preventing sexual transmission of HIV – as we have for the prevention of transmission of HIV through drug use- and renew the discourse about safer sex. Partner reduction and condom use already represent harm reduction approaches to dealing with risky sexual behaviours. Further multidisciplinary research is required to assess additional risk reduction strategies, including those observed on the ground.

### The control of HIV viral load: a new approach to sexual risk reduction?

The reduction of maternal viral load is the mainstay of efforts to prevent perinatal HIV transmission [22]. Viral load suppression has also been proposed as a strategy to reduce sexual transmission, in recognition of the strong association between viral load and risk of transmission. Early studies conducted in Rakai, Tanzania, among serodiscordant heterosexual couples before the antiretroviral era indicated that the HIV transmission rate is almost linearly associated with viral RNA levels in the plasma [23]. The advent of antiretroviral treatment (ART) spurred efforts to determine the impact of viral load suppression on HIV transmission.

Cohorts of sero-discordant couples have been observed throughout the ART era, and although the results are still sparse, they show that ART-induced viral load suppression is associated with reduced levels of HIV transmission [24]. In a Spanish cohort of serodiscordant couples, HIV transmission was reduced by 80% after introduction of higly active ART (HAART). In this study, transmission never occurred when virological suppression was achieved in the positive partner [25].

At the end of 2007, a statement issued by Swiss experts became the subject of much controversy [26]. Based on a review of Vernazza [27], the authors concluded that condom use may not be necessary in stable heterosexual serodiscordant couples in which virological suppression had been achieved in the positive partner for at least six months. Many concerns have been raised about this position. It has been pointed out that virological suppression in the blood is not necessarily associated with suppression in the genital fluids [28]. In addition, the data refer to stable heterosexual couples with no intercurrent sexually transmitted infection, which might lead to peaks in viral load. Despite these limitations, the results of the Swiss study may hold promise for sero-discordant couples, the population in which much transmission occurs in high prevalence countries [29]. It is not, however, clear how these results might apply to other populations at risk of HIV. ART may therefore be retained as a useful additional risk reduction strategy at the individual level, but more research is needed to determine its contribution to "combination prevention" within public health programmes.

The Swiss controversy gives prominence to the uneasy question of "when to start ART". The prospects of treating HIV-positive people for public health purposes, and not only to achieve benefits at an individual level, raises many complex issues. It is still an open question whether increasing the 200 CD4 threshold point globally recommended for initiating ART to the level recommended in most industrialized countries [30] would have an impact on HIV incidence. Recent modeling results provide a compelling argument that increasing ART use could lead to a dramatic reduction of HIV incidence, even when considering an increase in risky behavior [31]. This remains a priority area for further research.

### The impact of ART on condom use

Interestingly, in two separate studies, one conducted in Côte d'Ivoire [32], the other in Cameroon [33], we were able to demonstrate an independent positive effect of ART on the systematic use of condoms. In multivariate analysis, the odds ratio of systematic condom use was around twice as high among sexually active treated PLWHA than among untreated PLWHA. Such a result can probably be explained by the comprehensive care and support services provided to persons who are under treatment. In another study conducted in Kenya, PLWHA receiving ART were more likely to adopt safer behaviours than those not on treatment [34].

### The need for comprehensive services for PLWHA, including positive prevention

These data on the role of viral load on HIV transmission and behaviour change among PLWHA suggest that untreated PLWHA are more likely to transmit the virus, and underline the urgent need to reach this population with comprehensive prevention, care and support services. In all settings, PLWHA, while in good health, are of limited interest to health care workers, as they are not eligible for treatment, and, as a result, they tend to receive only limited psychosocial support services, if any at all. These services are critical to support safer sex practices. Treatment adherence support programmes have already been introduced in several settings and have been shown to be effective in promoting adherence when they are focused not only on treatment but also on the person and on all aspects of daily life [35].

For instance, Spire and others recently reported the results of a comprehensive programme for PLWHA in Phnom-Penh that was highly effective on treatment adherence. 95% of participants were fully adherent after two years of treatment [36]. Similar approaches could be helpful in designing behavioural interventions for individuals living with HIV, but not yet requiring treatment, which would empower them to adopt and maintain safer behaviours.

For PLWHA under treatment, all interventions that maintain long-term virological success are likely to reduce HIV transmission risk. However, to achieve this, a comprehensive approach that integrates all essential prevention, care and support services is required, to make the most of potential synergies. Several results obtained through multidisciplinary studies in France and Italy suggest that perception of treatment toxicity is a significant factor influencing adherence [37-39] as well as sexual risk behaviour [40,41]. The more side-effects PLWHA experience, the less adherent they are, and the less likely they are to use condoms systematically. This in turn negatively influences their quality of life [42]. Taking into account the patient's reported clinical outcomes could help when designing the best strategies to reduce viral load and risk behaviours.

This positive relationship between perception of health and consistent condom use has been confirmed in other studies, conducted among a cohort of PLWHA infected through drug use in France [43] and among PLWHA enrolled in the Agence nationale de recherches sur le sida et les hépatites virales (ANRS) Trivacan trial in Côte d'Ivoire [44]. In these two distinct populations, the same relationship between the capacity to consistently use condoms and the lack of perceived side-effects associated with ART was observed.

## Diversifying HIV testing approaches

### Shortening the duration of the "unknown infection" period

Shortening the duration of the "unknown infection" period should have an impact on HIV transmission. There are many benefits of an early diagnosis, in terms of life expectancy, but also quality of life [45]. From a public health point of view, increasing the proportion of PLWHA who know their status will also have a positive impact since those who know they are infected are more likely to adopt safer behaviours.

A meta-analysis indicated that the prevalence of unprotected intercourse was reduced by 53% in positive HIV persons in the USA who were aware of their status relative to those who were unaware [46]. There was a 68% reduction after adjusting the data of the primary studies to focus on unprotected intercourse with partners who were not already infected. In rural Zimbabwe, women who tested positive subsequently reported increased consistent condom use in their regular partnerships [47]. However, in many settings, HIV diagnoses occurs late, with negative consequences at both individual and public health levels.

### Diversifying and combining HIV testing approaches

In order to enable more people to learn their serostatus and to open access to HIV services to those in need, a range of HIV testing approaches need to be developed and implemented. Stigma, fear of receiving a HIV-positive status, lack of confidentiality, and long distances to dedicated HIV testing sites may represent barriers to the conventional voluntary counselling and testing approach. Provider-initiated HIV testing with an opt-out option has been widely debated for the last few years [48]. Data from high prevalence countries indicate that this approach to HIV testing in health care settings leads to significantly higher rates of detection of HIV infection and disease [49]. Alternative voluntary counselling and testing service delivery models, such as outreach through mobile vans, can also increase access to and uptake of testing [50]. In Morocco, a low HIV prevalence country, the community-based NGO Association de lutte contre le sida (ALCS) has been implementing for several years mobile testing strategies, and has found that these are more effective in reaching HIV-infected individuals than institutional voluntary counselling and testing approaches [51].

The added public health value of community HIV testing approaches needs to be further explored. Non-invasive methods based on rapid antibody assays can be readily carried out by non health-care professionals, and HIV screening services based on rapid HIV testing performed by community members has the potential to reach marginalized populations that are not otherwise served. In addition, the combination of peer-based counselling and rapid testing could represent an interesting preventive strategy for highly exposed individuals who need repeated HIV tests but who have difficulty in availing of health-facility based voluntary counselling and testing services.

Repeated testing may be useful to reach individuals during the period of primary HIV infection when risk of ongoing transmission is particularly high, due to sharply elevated viral load [52]. The role of primary infection in the epidemic dynamics was highlighted last year in a Canadian study. Phylogenetic analyses suggested that early infection accounted for approximately half of onward transmissions over a period of about a year in the city of Montreal [53]. These data suggest that sero-ignorance during the early stages of the disease may make a significant contribution to ongoing transmission.

Strategies to increase access to HIV testing should not only facilitate entry into the health system and support community-based approaches, but should also make provision for repeat tests, especially for people who live with risk. The earlier people know about their infection, the less likely they are to transmit the virus.

## Dealing with stigma and discrimination

### Stigma and discrimination fuel the epidemic

There is growing evidence that stigma and discrimination contribute to risky behaviours in both HIV positive and HIV negative individuals. In several parts of the world, the fear of stigma and discrimination is associated with lower uptake of HIV testing and less willingness to disclose positive results [54]. Recent data from the French ANRS VESPA study based on a large representative sample of PLWHA shows that perceived stigma is associated with risky health behaviours such as non-adherence [55] and unprotected sex [56]. More specifically a relationship between inconsistent condom use and experience of discrimination was found among heterosexuals and injecting drug users. Multivariate analysis shows that discrimination from one's closest friends and relatives was an independent factor associated with non- systematic use of condoms.

Another problem is the double stigma that affects some groups, such as injecting drug users, sex workers and men who have sex with men. These groups already face a higher risk of HIV infection, associated with specific behaviours and practices, for which they are blamed. In addition, they are ostracized independently of HIV infection. This double stigma probably contributes to the high HIV burden in these groups, as stigma and fear of stigma significantly constrain access to information and to services. Yet, these groups are in greatest need of comprehensive HIV prevention, care and treatment services. Many studies have compared HIV prevalence among MSM with prevalence in the general population. In most countries of the world, HIV prevalence is consistently higher among MSM. Odds ratios for HIV infection in MSM are elevated across prevalence levels by country and decrease as general population prevalence increases, but remain 9-fold higher in medium-high prevalence settings [57]. Similar findings are reported among sex workers and drug users, who are consistently found to have much higher prevalence rates than other segments of the population.

### A triple therapy against stigma and discrimination

We propose an effective "triple therapy" approach to fight against stigma and discrimination. The proposed regimen would include the following: first, fight for greater acceptance of PLWHA; second, improve relevant laws and policies, and third, involve prevention users – *work with people rather than for people*.

Greater acceptance of HIV in our society would help people break the secret and help them to disclose their status without fear. The experience of AIDES in France and also of several of its African partners, is that strengthening the social positions of PLWHA reinforces the collective ability to talk about HIV. It also induces changes in the way society regards PLWHA. Of course, the ability to talk is associated with the ability to listen, which underlines the importance of public action, based for instance on campaigns featuring personalities and opinion leaders. The use of political leaders in such campaigns is also of interest; such as those used in France during the 2007 presidential election [58]. The goal of these campaigns, which have been very popular, is to make people reflect on how HIV status impacts attitudes. Associated with other measures, such as public testimonies of PLWHA, and meaningful engagement of PLWHA in the design, implementation and evaluation of HIV programmes, they can contribute to changing the representation of HIV in the general public.

The improvement of laws and policies should serve to protect PLWHA and all vulnerable groups. Prevailing iniquitous measures undermine the response to AIDS. Laws which discriminate against or criminalize drug users, men who have sex with men, sex workers, and immigrants are likely to compromise their access to, and utilization of HIV prevention, care and treatment services, which in turn is likely to contribute to the spread of the epidemic. It is encouraging that some Latin American countries have launched policies and programs focused on reducing homophobia. Such measures urgently need to be introduced in other settings, particularly in Africa where community-based HIV prevention work can be dangerous for both the service providers and clients. This is exemplified by the recent arrest of gay prevention activists in some African countries. Similarly, the repression of drug users in several countries runs counter to public health interests. Changes will only be possible if international institutions, especially financial backers, step up pressure on governments to guarantee a rights-based approach to public health. Changes will also be brought about by involving communities affected by HIV in the process of decision making.

Community mobilization among PLWHA has been shown to be a driving force in increasing access to treatment. To improve HIV prevention efforts, HIV-positive people, those who are most exposed to infection and affected communities need to mobilize together. There must be a real effort to ensure that those living with HIV are truly involved by occupying key positions in non governmental organizations. The mobilization of people who are "sero-concerned" is essential as the professional response will never be sufficient.

For the last 25 years, HIV prevention uptake has improved through community mobilization and peer support, leading to the empowerment of those who are marginalized and most at risk [59]. People who are only "experts based on their life experience" [60] have run prevention programmes for gay men, conceptualized harm reduction approaches for drug users, and reached out with HIV prevention services to marginalized groups such as migrant women.

The mobilization of sex workers all over the world has led to successes in prevention programmes. The Sonagachi project in India, run by and for sex workers, has resulted in impressive coverage rates with HIV and other services for sex workers in the state of West Bengal and HIV prevalence rates have remained low in these communities [61]. In Santo Domingo, interventions combining support for sex worker solidarity and changes in government policy are showing positive effects. In Paris, the PAST project has mobilized sex workers and transgenders to claim their rights and obtain services.

Finally, a breakthrough in the field of community mobilization is the emergence of gay men in an African context. Until recently MSM were not counted and ignored by African and international policy-makers. AIDES has recently supported the mobilization of gay Africans, and has found that MSM are not so few, they are visible, they want to contribute to the public health policies and can become community health actors despite homophobic environments [62].

## The way forward: investing in community mobilization

HIV prevention can work when it reflects the comprehensive needs of people. Our experience with community mobilization in concentrated epidemics is that it is an essential component of the response. In generalized epidemics, more research and experience are needed to understand how to mobilize those most at risk. Nonetheless, the empowerment of communities remains a global imperative and challenge. It requires real empowerment of PLWHA as well as the empowerment of marginalized and stigmatized populations. The key message is to involve lay-men and women in public health action.

With this aim, four non-governmental organizations that privilege community involvement and recognize acquired expertise have decided to create a new international structure called PLUS. Its goal is to enable the voices of sero-concerned people to reach and influence international policy makers, by enhancing the global visibility of community commitment in the fight against AIDS and promoting community-based research.

## Competing interests

The authors declare that they have no competing interests.

## Authors' contributions

BS conceived the manuscript. BS and ID reviewed the literature and wrote the manuscript; HH participated to the writing and provide reports on community HIV testing. All authors read and approved the final manuscript.
